# A Case of Abscess Formation after Radiofrequency Ablation for Early Breast Cancer

**DOI:** 10.70352/scrj.cr.26-0153

**Published:** 2026-05-09

**Authors:** Kenjiro Jimbo, Takashige Tsuji

**Affiliations:** Department of Breast Surgery, Shonan Kamakura General Hospital, Kamakura, Kanagawa, Japan

**Keywords:** breast cancer, radiofrequency ablation, abscess, delayed complication, case report

## Abstract

**INTRODUCTION:**

Breast-conserving surgery with whole-breast irradiation is the standard local treatment for early-stage breast cancer. Recently, less invasive approaches have gained attention with advances in imaging and ablative technologies. Radiofrequency ablation (RFA) induces thermal coagulative necrosis and has been explored as a potential alternative for selected small breast tumors, with favorable oncological and cosmetic outcomes reported. However, data regarding infectious complications remain limited. Because RFA creates devitalized tissue, secondary infection and abscess formation may occur. We report a rare case of delayed abscess formation after breast RFA that required surgical management.

**CASE PRESENTATION:**

A 58-year-old woman with cT1bN0M0 invasive ductal carcinoma of the left breast underwent RFA combined with sentinel lymph node biopsy. Adjuvant endocrine therapy and whole-breast irradiation were subsequently administered. Four months after RFA, MRI and vacuum-assisted biopsy confirmed complete tumor ablation. Six months after the procedure, the patient developed progressive swelling, erythema, and tenderness of the left breast. Despite systemic antibiotic therapy, symptoms persisted. Contrast-enhanced CT and ultrasonography revealed fluid accumulation within the ablation zone, consistent with abscess formation. Surgical incision and drainage with debridement were performed. The patient recovered gradually, and complete wound healing was achieved 2 months after the intervention.

**CONCLUSIONS:**

We report a rare case of delayed abscess formation following breast RFA that required surgical management. As RFA becomes more widely implemented, awareness of potential late infectious complications and prompt intervention are essential for ensuring patient safety.

## Abbreviation

RFA
: radiofrequency ablation

## INTRODUCTION

Breast-conserving surgery followed by whole-breast irradiation has long been established as the standard local treatment for early-stage breast cancer. However, the pursuit of less invasive local therapies has intensified over the past 2 decades, driven by patient preference, advances in imaging guidance, and the development of ablative technologies. Minimally invasive local treatments, including RFA, have been proposed as alternatives to surgical resection in carefully selected patients with small, localized breast tumors.^1)^ Several prospective studies have demonstrated acceptable local control and cosmetic outcomes after breast RFA. Because RFA induces coagulative necrosis of breast tissue, there is a theoretical risk of secondary infection. Severe infectious complications requiring surgical management have rarely been reported. We present a rare and instructive case of a post-RFA abscess that required surgical intervention.

## CASE PRESENTATION

**Fig. 1:**
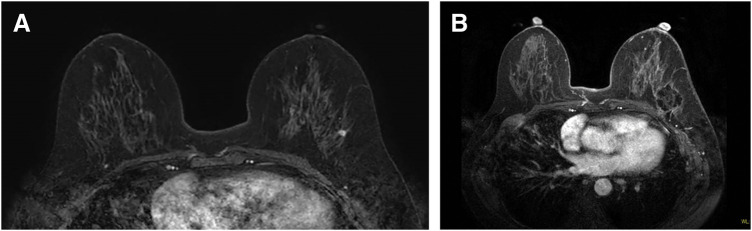
MRI at diagnosis and after RFA. (**A**) Pretreatment breast MRI revealed a localized tumor in the C area of the left breast. (**B**) Three months after RFA, MRI revealed a well-defined hypointense area corresponding to the ablation zone with nonviable internal architecture, and no residual tumor was detected. RFA, radiofrequency ablation

**Fig. 2:**
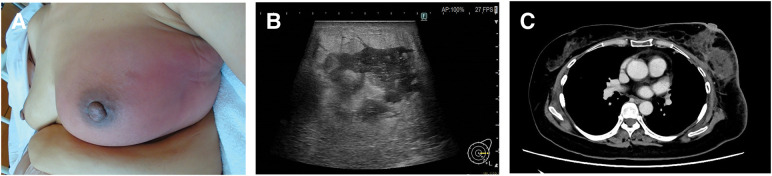
Clinical and imaging findings (**A**) Despite antibiotic therapy, the left breast exhibited marked swelling, erythema, and warmth. Prominent swelling was observed at the RFA-treated site in the C area. (**B**) Ultrasonography revealed fluid collection at the RFA site, suggesting abscess formation. The surrounding subcutaneous tissue exhibited inflammatory edematous changes. (**C**) Contrast-enhanced CT demonstrated fluid collection consistent with abscess formation centered at the RFA ablation site. RFA, radiofrequency ablation

**Fig. 3:**
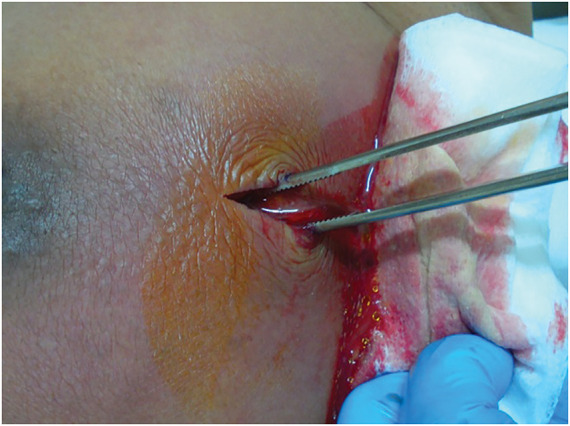
Intraoperative findings. Incision and drainage under local anesthesia resulted in the evacuation of a large amount of purulent material containing necrotic tissue.

**Fig. 4:**
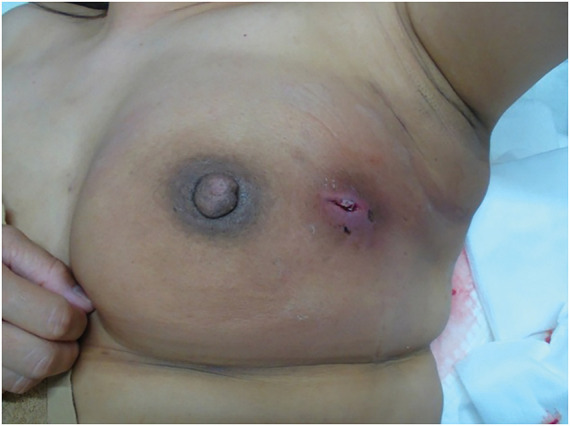
Clinical appearance (11 days of drainage). Marked improvement in inflammatory findings was observed.

A 58-year-old woman noticed a palpable mass in her left breast and visited a local clinic. After a core needle biopsy, she was diagnosed with left-sided invasive ductal carcinoma (cT1bN0M0, cStage IA; **Fig. 1A**). The tumor was hormone receptor-positive and human epidermal growth factor receptor 2-negative. Based on imaging findings, the lesion was considered suitable for RFA. The patient subsequently underwent RFA of the breast cancer lesion combined with a sentinel lymph node biopsy. No nodal metastasis was identified. Postoperatively, she received adjuvant endocrine therapy and whole-breast irradiation (53.2 Gy in 20 fractions), which was completed approximately 2 months after RFA. Four months after RFA, MRI revealed a well-defined hypointense area corresponding to the ablation zone, and no residual tumor was detected (**Fig. 1B**). A vacuum-assisted biopsy (VAB) of the ablation site was performed to evaluate residual disease. Histopathological examination revealed no viable tumor cells, confirming complete local tumor ablation. Six months after RFA, the patient developed diffuse swelling, erythema, and tenderness involving the entire left breast (**Fig. 2A**). Breast cellulitis associated with radiation dermatitis was suspected, and systemic antibiotics were administered. Despite 10 days of conservative antibiotic therapy, her symptoms did not improve. Contrast-enhanced CT and ultrasonography revealed fluid accumulation within the center of the RFA-treated area (**Fig. 2B** and **2C**). Based on these findings, a diagnosis of delayed postoperative abscess at the RFA ablation site was made. Surgical incision and drainage were performed. Purulence containing necrotic tissue was removed as thoroughly as possible. The abscess cavity was irrigated with normal saline (**Fig. 3**). Microbiological cultures were not obtained from the purulent fluid. Swelling and erythema gradually improved following surgical drainage (**Fig. 4**). Complete healing of the wound was achieved approximately 2 months after the intervention.

## DISCUSSION

Early feasibility and retrospective studies demonstrated that RFA could achieve acceptable local tumor control with favorable cosmetic outcomes in selected patients with early-stage disease.^2–7)^ Systematic reviews have also highlighted RFA as an emerging nonsurgical ablative approach for breast cancer, emphasizing its reduced invasiveness.^8)^ In particular, prospective studies in Japan, including the PO-RAFAELO and RAFAELO trials, reported low rates of ipsilateral breast tumor recurrence and acceptable safety profiles when RFA is used as definitive local therapy without routine surgical excision.^4–6)^ Nevertheless, most published studies have primarily focused on oncological efficacy and cosmetic outcomes, whereas detailed analyses of adverse events remain limited. Common early adverse events after RFA for breast cancer include local pain, skin burns, and minor bleeding-related events such as subcutaneous hemorrhage and hematoma. Most reported complications are mild and self-limiting, with low overall incidence rates.^8–10)^ Zhou et al. reported that US-guided percutaneous thermal ablation for breast tumors was associated with a low overall complication rate, and no major life-threatening adverse events were observed in their clinical series.^10)^ Prospective studies in Japan, including the PO-RAFAELO and RAFAELO trials, also recorded low rates of serious short-term complications, with few, if any, grade 3 or higher adverse events, supporting the favorable safety profile of RFA as a minimally invasive local treatment compared with conventional breast-conserving surgery.^4–6)^ However, because RFA intentionally creates a volume of devitalized tissue within the breast, there is a theoretical risk of secondary infection and abscess formation. Although infectious complications after breast RFA have been reported, the incidence of abscess formation has not been well established, and cases requiring surgical intervention appear to be uncommon in the current literature.^9,10)^ Specific preventive criteria have not been clearly defined, and potentially modifiable factors should be considered. These include invasive procedures within the ablation zone, such as VAB. In Japan, VAB is required as part of the standard post-RFA assessment. Although essential for confirming the absence of residual tumor, it may theoretically increase the risk of bacterial introduction into the avascular necrotic ablation zone; therefore, strict aseptic technique is required. Other factors include careful assessment of patients with uncontrolled infection-related conditions and ensuring an adequate tumor–skin distance to reduce the risk of infection via the skin tract. Necrotic tissue can act as a nidus for bacterial colonization, and once infection develops, antibiotic penetration into the avascular ablation zone may be limited. In the present case, the development of infection was likely multifactorial. Patient-related factors, such as comorbidities and breast characteristics, may influence the risk of infection after RFA; however, their impact has not been well established. While appropriate patient selection is important, the present case alone does not allow for the identification of specific risk factors for abscess formation. Overall complication rates of breast RFA have been reported to be low, but abscess formation represents a clinically meaningful adverse event that can significantly affect patient outcomes and quality of life. Ultimately, surgical drainage and debridement were required to achieve infection control, underscoring the limitations of nonsurgical management in such scenarios. A limitation of this case is the absence of microbiological confirmation, as bacterial cultures were not obtained at the time of drainage. Nevertheless, the clinical and imaging findings strongly supported the diagnosis of an abscess. In this case, VAB of the ablation site was performed 4 months after RFA, followed by abscess formation 2 months later. While a direct causal relationship cannot be confirmed, VAB may have contributed to bacterial introduction into the avascular necrotic ablation zone. Therefore, strict aseptic technique and close follow-up are warranted when performing invasive procedures at the ablation site. Radiotherapy may also have contributed to delayed abscess formation, as it can impair tissue vascularity and healing. In the present case, radiation-induced changes combined with persistent necrotic tissue after RFA may have predisposed the patient to infection. Previous breast RFA studies often incorporated delayed surgical excision of the treated lesion to confirm complete tumor ablation.^3,7)^ This practice might have removed necrotic tissue before infectious complications could develop, thereby leading to an underestimation of the true incidence of delayed abscess formation. By contrast, reports focusing on exclusive percutaneous RFA without planned surgical excision remain relatively scarce, and long-term safety data regarding infectious complications are still evolving.^4–6)^ As RFA transitions into routine practice, heightened awareness of potential late complications is essential. Careful patient selection, as discussed above, strict adherence to aseptic technique, consideration of periprocedural antibiotic prophylaxis, and close post-treatment surveillance are critical to minimize the risk of severe infection. Moreover, prompt imaging evaluation should be considered when inflammatory changes persist or worsen after RFA. This case provides important real-world safety data on breast RFA and highlights that delayed refractory abscess formation can occur and may require surgical intervention, even after complete tumor ablation. As breast RFA has recently been approved for insurance coverage in Japan and is expected to be more widely implemented in routine clinical practice, real-world case accumulation will help refine patient selection and post-treatment surveillance.

## CONCLUSIONS

We report a rare case of delayed abscess formation following breast RFA that required surgical management. As RFA becomes more widely implemented, awareness of potential late infectious complications and prompt intervention are essential to ensure patient safety.
